# Occupational health in times of pandemic

**DOI:** 10.47626/1679-4435-2020-183

**Published:** 2021-02-11

**Authors:** Andrea Franco Amoras Magalhães

**Affiliations:** Editor-in-chief, *Brazilian Journal of Occupational Medicine*

In March of this year, the World Health Organization (WHO) declared the COVID-19 outbreak a pandemic, a disease caused by the 2019 novel coronavirus (SARS-CoV-2) - a virus with a major impact on population health and on the economy of countries.

Also in March, the Brazilian Ministry of Health declared a state of community transmission of the novel coronavirus throughout the national territory and recommended that all health facilities should perform syndromic diagnosis for the management of suspected cases of COVID-19, regardless of the etiologic agent of the disease.

In view of this reality, the occupational health departments of companies and institutions have developed several actions to protect their workforce, aimed at preventing contamination by the coronavirus in the workplace always based on the latest guidelines issued by health authorities to confront the novel coronavirus.

In this scenario, it is worth noting the development of protocols with measures to prevent SARS-CoV-2 infection, with the purpose of monitoring staff health especially during the pandemic. Assessing the effects of the pandemic on workers providing essential services, especially those working in businesses that have not closed and are gradually resuming full production, is essential for early detection of the disease and prevention of outbreaks within the workplace.

However, because this is a novel virus, whose scientific studies are continuously updated, researchers and test developers keep seeking innovative solutions to reduce the time to obtaining COVID-19 test results. These measures are very important for the maintenance of the workforce and business services as we await that science will provide us with a safe and effective vaccine against the disease.

Occupational medicine has shown its strength, resilience, creativity, and bravery in facing the pandemic. We are proud of our performance and have demonstrated our importance for employment protection and economic recovery. Our journal reflects, through the studies conducted, the concerns and main lines of care of occupational health.

Although COVID-19 has led to an atypical year, it is refreshing to see that professionals continue their research even when beset by problems and adversities, in the search to overcome them. We are very pleased to present you with another issue of our journal. Hope you enjoy reading this issue!

Andrea Franco Amoras MagalhãesEditor-in-chief, *Brazilian Journal of Occupational Medicine*

